# Co-Occurring CSF3R W791* Germline and Somatic T618I Driver Mutations Induce Early CNL and Clonal Progression to Mixed Phenotype Acute Leukemia

**DOI:** 10.3390/curroncol29020068

**Published:** 2022-02-01

**Authors:** Franziska C. Adam, Jakub Szybinski, Jörg P. Halter, Nathan Cantoni, Friedel Wenzel, Katharina Leonards, Sime Brkic, Jakob R. Passweg, Ivo Touw, Julia E. Maxson, Sara C. Meyer

**Affiliations:** 1 Division of Hematology, University Hospital Basel, CH-4031 Basel, Switzerland; franziska.adam@usb.ch (F.C.A.); joerg.halter@usb.ch (J.P.H.); katharina.leonards@usb.ch (K.L.); jakob.passweg@usb.ch (J.R.P.); 2 Department of Biomedicine, University Hospital Basel and University of Basel, CH-4031 Basel, Switzerland; jakub.szybinski@unibas.ch (J.S.); sime.brkic@unibas.ch (S.B.); 3 Kantonsspital Aarau, CH-5001 Aarau, Switzerland; nathan.cantoni@ksa.ch; 4 Institute of Medical Genetics and Pathology, University Hospital Basel, CH-4031 Basel, Switzerland; friedel.wenzel@usb.ch; 5 Department of Hematology, Erasmus University Medical Center, 3015 CN Rotterdam, The Netherlands; i.touw@erasmusmc.nl; 6 Knight Cancer Institute, Oregon Health & Science University, Portland, OR 97239, USA; maxsonj@ohsu.edu

**Keywords:** CSF3R, clonal evolution, chronic neutrophilic leukemia, mixed phenotype acute leukemia

## Abstract

Chronic neutrophilic leukemia (CNL) relates to mutational CSF3R activation with membrane proximal *CSF3R* mutations such as T618I as driver mutations, but the significance of truncating mutations is not clarified. In CNL, concomitant mutations promote disease progression, but insight into longitudinal acquisition is incomplete. In this study, we investigated the role of co-occurring germline and somatic *CSF3R* mutations in CNL, and assessed the impact of clonal evolution on transformation to acute leukemia. We employed sequential next generation sequencing and SNP array karyotyping to assess clonal evolution in CNL of early manifestation age based on a 33-year-old patient. Germline vs. somatic mutations were differentiated using a sample from the hair follicle. To investigate a potential predisposition for CNL development and progression by germline *CSF3R*-W791*, allelic localizations were evaluated. We detected a somatic *CSF3R*-T618I mutation at 46% variant allele frequency (VAF) at the time of CNL diagnosis, which co-occurred with a *CSF3R*-W791* truncation at 50% VAF in the germline. Evaluation of allelic localization revealed *CSF3R*-T618I and W791* on the same allele. A concomitant *ASXL1* mutation at 39% VAF increased to 48% VAF upon transformation to mixed phenotype acute leukemia (MPAL), which has both myeloid and lymphoid features. Clonal evolution further involved expansion of the *CSF3R* double-mutant clone to 90% VAF via copy neutral loss of heterozygosity on chromosome 1p and the emergence of a *RUNX1* mutant subclone. Allogeneic transplantation induced complete remission. This study highlights that CNL not only transforms to AML but also to MPAL. The molecular evolution is especially interesting with a *CSF3R*-W791* mutation in the germline and acquisition of *CSF3R*-T618I on the same allele compatible with increased susceptibility for mutation acquisition facilitating *RUNX1*-related clonal transformation.

## 1. Introduction

Chronic neutrophilic leukemia (CNL) is a myeloproliferative neoplasm characterized by the overproduction of neutrophils and activating mutations in *CSF3R*, the receptor for colony stimulating factor 3 (GCSF). Presentation is heterogeneous ranging from asymptomatic clonal neutrophilia to severe constitutional symptoms, splenomegaly, bleeding diathesis and an inherent risk for transformation to acute myeloid leukemia (AML) [1]. Activating point mutations in *CSF3R*, most prevalently *CSF3R* T618I, were discovered in 2013 and recognized as driver mutation in a majority of CNL patients [2,3]. These activating mutations localize to a membrane proximal, extracellular portion of the CSF3R and induce ligand independent receptor activation resulting in constitutive activation of the JAK-STAT signaling pathway. Their essential role for CNL pathogenesis has led to the implementation as key genetic biomarker into the WHO diagnostic criteria for CNL, which greatly facilitated CNL diagnosis along with neutrophilia and hypercellular marrow with increased granulopoiesis [4,5]. In line with JAK-STAT pathway activation, efficacy of the JAK1/2 inhibitor ruxolitinib has been demonstrated in case reports and a clinical trial for patients with CNL and atypical CML, a related myeloproliferative disorder [2,6]. Rarely, *CSF3R* activating mutations such as T618I, T640N and N610H have also been detected in germline in individuals with congenital neutrophilia [7,8,9].

Genetic characterization of *CSF3R* in CNL has also revealed nonsense or frameshift mutations in more distal, intracellular portions of CSF3R resulting in receptor truncation [2]. It has been shown that this second class of *CSF3R* mutations increases cell surface expression of CSF3R, mediating ligand hypersensitivity [10]. Notably, a minority of *CSF3R*-T618I mutant CNL cases also show a concomitant *CSF3R* truncation mutation. It is not entirely clear how activating and truncating *CSF3R* co-mutations alter progression to acute leukemia, although truncating mutations can enhance *CSF3R*-T618I-mediated growth in some in vitro assays [11]. Germline *CSF3R* cytoplasmic truncation mutations have not been reported thus far.

Transformation to acute leukemia is reported in 10–21% of CNL patients and median time to transformation is approximately 21 months [1]. Mutations in the epigenetic regulator *ASXL1*, which are frequently detected along with *CSF3R* mutations at CNL diagnosis, confer adverse prognosis and increased risk of transformation [12]. However, the molecular dynamics of clonal evolution and progression of CNL to AML is incompletely understood [13]. Comprehensive genomic and transcriptomic profiling of CNL patients revealed multiple co-occurring mutations, which are enriched in CNL, including *ASXL1*, *SRSF2*, *SETBP1*, *TET2*, *EZH2*, *U2AF1* and others [13]. To date, specific insight into clonal evolution and transformation of CNL patients is incomplete and particularly challenging given the rarity of this disease. Detailed molecular characterization is warranted to enable improved prognostication and inform novel therapeutic approaches.

*CSF3R* truncation mutations are also prevalent in patients with severe congenital neutropenia (SCN), a condition with heterogenous basis often related to mutations in the *ELANE* gene encoding neutrophil elastase [14]. While SCN patients are often managed by long-term administration of G-CSF, the presence of *CSF3R* truncation mutations associated with increased risk of progression from SCN to AML [15,16]. Studies of SCN clonal evolution have typically shown the emergence of subclones with *RUNX1*, *ASXL1* or *SUZ12* mutations [17]. More rarely, acquisition of activating point mutations in *CSF3R* as e.g., T618I has also been seen [18]. Thus, *CSF3R* truncating and activating mutations may co-occur in SCN similarly to CNL, but while activating *CSF3R* mutations are present in the vast majority of CNL patients with a minority co-carrying truncation mutants, truncating *CSF3R* mutations prevail in SCN [18]. This may in part relate to the differentiation potential of the two classes of mutations, while *CSF3R*-T618I seems to maintain full differentiation potential, *CSF3R* truncating mutations impair differentiation [19]. Here we investigate the role of co-occurring *CSF3R* germline and somatic mutations and clonal evolution in CNL for transformation to acute leukemia based on a patient with early onset CNL. We report for the first time CNL transformation to acute leukemia of mixed phenotype (MPAL type) in the setting of a germline *CSF3R*-W791* truncation mutation located on the same allele as *CSF3R*-T618I, suggesting increased susceptibility for *RUNX1*-related clonal transformation.

## 2. Materials and Methods

### 2.1. Mutational Analysis by Next Generation Sequencing

**Figure 1:**
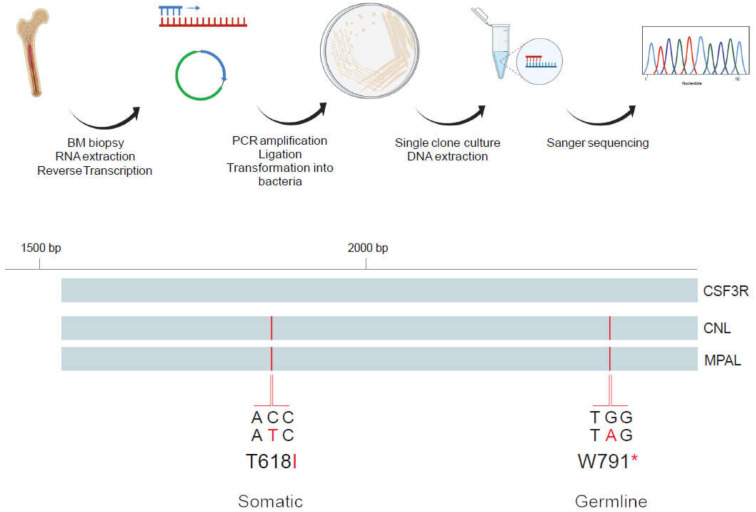
Allelic localization of germline *CSF3R* W791* and acquired *CSF3R* T618I in CNL and clonal progression to acute leukemia of mixed phenotype (MPAL type). Patient bone marrow cDNA from the *CSF3R* gene locus encompassing both T618I and W791* was amplified by PCR and Sanger sequencing of plasmid-emergent colonies to evaluate allelic localization (upper panel). *CSF3R* reference sequence (bottom panel, top line) was aligned with patient-derived DNA sequences at the stage of CNL (bottom panel, middle line) and of mixed phenotype acute leukemia (MPAL, bottom panel, bottom line). Sequence analyses demonstrated co-localization of T618I and W791* mutations (red vertical lines) *in cis* on the same allele at CNL and MPAL stages.

Mutational status was assessed using a 39-gene targeted AmpliSeq NGS assay covering full genes including splice sites covering *ABL1*, *ASXL1*, *BCR*, *BRAF*, *CALR*, *CBL*, *CEBPA*, *CHEK2*, *CSF3R*, *DNMT3A*, *EGLN1*, *EPOR*, *ETNK1*, *ETV6*, *EZH2*, *FLT3*, *GATA2*, *IDH1*, *IDH2*, *JAK2*, *KIT*, *KRAS*, *MPL*, *NF1*, *NPM1*, *NRAS*, *PDGFRA*, *PDGFRB*, *PTPN11*, *RUNX1*, *SETBP1*, *SF3B1*, *SH2B3*, *SRSF2*, *TET2*, *TP53*, *U2AF1*, *VHL*, *ZRSR2*. A total of 60 ng DNA extracted from whole bone marrow cells was amplified with 15 PCR cycles during automated library preparation with the Ion AmpliSeq Kit for Chef DL8 (Cat. A29024) on the IonChef instrument. Library pools were quantified by qPCR using the Ion Universal Quantitation Kit (Cat. A26217) and 30 pmol of the pool was sequenced on 530 Chips on an Ion S5XL sequencer. Sequences were aligned to the human reference genome GRCh37/hg19 and analyzed with IonReporter version 5.12 with a sensitivity limit of variant allelic frequency of 2–5%. For analysis of germline mutations, 60 ng DNA extracted from hair follicles was processed analogously as described above. Variant allele frequencies were plotted with GraphPadPrism9. Figure 1 was created with Biorender software (Biorender.com, last accession date 1 November 2021)

### 2.2. Analysis of Allelic Localization of CSF3R Mutations by Sequencing of Plasmid DNA Clones

The *CSF3R* gene region spanning both activating T618I and truncating W791* mutations was amplified by PCR from 4µg of bone marrow derived cDNA using *CSF3R* forward (5′-ACCATGGGACCCTCCCAGCA-3′) and reverse primers (5′-AAGCTCCCCAGCGCCTCCAT -3′) and Platinum^TM^ *Taq* DNA Polymerase (Invitrogen, Waltham, MA, USA). Resulting PCR products were separated by agarose gel electrophoresis and gel extraction of PCR products of desired band-size was performed using Qiaquick Gel Extraction kit (Qiagen, Venlo, The Netherlands). Amplified CSF3R fragments were cloned into pCR4-TOPO vector and transformed into One Shot^TM^ TOP10 *E. coli* bacteria (Invitrogen), which were plated on agar plates supplemented with ampicillin and cultured overnight at 37 °C. Single bacteria colonies were picked and expanded in LB-medium cultures. DNA was extracted by Mini-preparation of plasmid DNA (Qiagen), processed by restriction enzyme digestion with EcoRI HF^®^ (New England BioLabs, Ipswich, MA, USA), run on agarose gel and analyzed for presence of the CSF3R insert. TOPO M13 reverse primer was added to DNA from clones with *CSF3R* insert and 6 clones per disease stage were subjected to Sanger sequencing. Resulting sequences were aligned to *CSF3R* transcript variant 1 (NCBI Reference Sequence: NM_000760.4) and analyzed using Benchling Biology Software (2021), retrieved from https://benchling.com (last accession date 16 June 2021).

### 2.3. SNP Array Karyotyping

DNA was extracted from bone marrow by MagnaPur (Roche, Basel, Switzerland) or Prepito-D system (PerkinElmer, Waltham, MA, USA) and DNA blood 600 kit (Chemagen, Baesweiler, Germany) and hybridized to Cytoscan HD array (Affymetrix, Santa Clara, CA, USA) according to manufacturer’s instructions. Data were analyzed using the Chromosome Analysis Suite (ChAS) software (Affymetrix). Copy number variants (CNV) were considered with a size above 100 kb; copy neutral loss of heterozygosity (CN-LOH) >5 Mb and extending to telomeres is defined as acquired abnormality. All CNV and CN-LOH fulfilling these criteria were considered for this analysis and validated by visual inspection as well as annotated for size, position and location of genes based on the human genome version 19 (hg19) of USCS Genome Browser.

## 3. Results

### 3.1. Early Onset CNL Demonstrates Progression to Mixed Phenotype Acute Leukemia

CNL was diagnosed in a 33-year-old male in the absence of comorbidities or neoplastic disorders in his relatives according to WHO criteria (Table 1). Initial presentation was with symptomatic splenomegaly, shortness of breath, elevated white blood cell count (WBC) of 53 × 10^9^/L with pronounced neutrophilia of 88% and mild anemia. Bone marrow was hypercellular with greatly increased and maturing granulopoiesis. *BCR-ABL1* fusion was negative while CSF3R mutations in CNL had yet to be discovered at the time of CNL diagnosis.

Cytoreductive treatment with hydroxyurea led to hematologic remission. Upon progressive neutrophilia and splenomegaly 5 years later, JAK1/2 inhibitor therapy with ruxolitinib was initiated resulting in a favorable clinical response and was intermittently switched to pegylated interferon alpha given the patient’s wish to father children.

After a stable ten-year course, sudden clinical deterioration occurred, and diagnosis of acute leukemia was made. Leukemic blasts with extensive marrow infiltration were partially positive for cytochemical peroxidase staining (20–30%), while Auer rods were absent, and immunophenotypic characterization confirmed co-existence of a myeloid CD34+, CD117+/−, HLA-DR+, cyMPO+/−, CD13+, CD33+, CD36+, CD71+ blast population negative for CD3-, cyCD3, CD19 and CD79a alongside a CD19+, CD22+/−, cyCD79a+, nuTDT+ und cyCD3- B-lymphoid blast population consistent with mixed phenotype acute leukemia (MPAL type B/myeloid) according to WHO criteria. To our knowledge, this represents the first report of CNL transforming to acute leukemia of MPAL type. After induction chemotherapy and consolidation by allogeneic hematopoietic stem cell transplantation (HSCT), the patient achieved continuous complete remission until the last follow-up, 24 months post-transplant. The patient’s mother and father have deceased (due to non-hematologic disorders), but no specific information is available on hematologic aberrancies in the patient’s parents or six siblings.

### 3.2. Characterization of CSF3R Mutations Reveals W791* in Germline and in Cis with Somatic T618I

Given the particular features of transformation to acute leukemia in this early onset CNL, we hypothesized that specific genetic factors could cooperate to promote emergence of CNL at early age and ultimately promote progression to acute leukemia. Thus, we investigated *CSF3R* gene mutations as well as the dynamics of clonal evolution by a sequential NGS approach using a 39-myeloid gene panel routinely used in our department. We detected the *CSF3R* T618I mutation at a variant allele frequency (VAF) of 46% and *ASXL1* C759fs mutation at 39% at CNL diagnosis in line with a high prevalence of *ASXL1* mutations in CNL. Of note, we identified a co-occurrent *CSF3R* W791* truncation mutation at 50% VAF suggestive of germline origin. Analysis of germline DNA from hair follicle confirmed *CSF3R* W791* truncation mutation as a likely germline variant at 50% VAF (Supplementary Figure S1). This finding adds new insight into CSF3R biology and contrasts with previous reports of rare germline *CSF3R* mutations identified in individuals with congenital neutrophilia, which so far exclusively represent *CSF3R* missense but not truncation mutations.

We next sought to establish whether a *CSF3R* germline truncation mutant could be involved in setting the stage and contribute to predisposition for CNL and leukemia development. To address this aspect, we evaluated the allelic localization of the co-occurring *CSF3R* auto-activating and truncation mutations. Patient-derived bone marrow DNA was amplified using a primer set that encompasses both mutations of interest, and was processed using a cloning approach and subjected to classical Sanger sequencing to assess for co-localization of the *CSF3R* T618I and W791* stop mutations on the same allele. Processed clones from CNL diagnosis revealed T618I and W791* mutations *in cis* on the identical allele (Figure 1). This was confirmed in leukemic blasts at the time-point of transformation with both mutations consistently located *in cis* in 6/6 analyzed clones. This finding is compatible with the notion of the *CSF3R* W791* germline variant mediating a potential predisposition for the acquisition of the *CSF3R* T618I driver mutation, thus facilitating early onset of CNL.

### 3.3. Clonal Progression Driven by CSF3R LOH and RUNX1 Mutation

**Figure 2:**
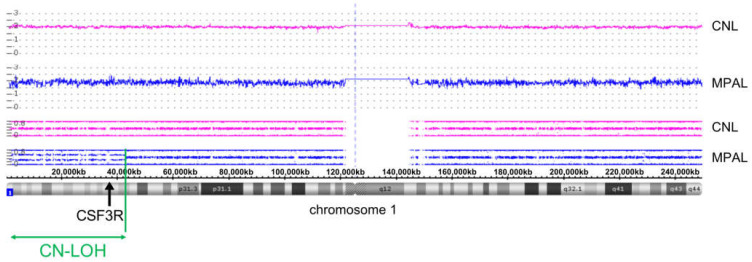
Copy neutral loss of heterozygosity (CN-LOH) reflected by SNP array karyotyping upon CNL progression to acute leukemia of mixed phenotype (MPAL type). Purple color represents DNA from CNL phase, blue color represents DNA from time-point of transformation to acute leukemia of MPAL type. Upper panels show a smooth signal distribution for DNA from CNL and MPAL stage indicating continuous and unchanged copy number of 2 for both samples. Middle panels show B-allele frequency (BAF) distributions with DNA from CNL phase (purple) with normal “three-line pattern” indicating no LOH, and DNA from MPAL phase (blue) with “four-line pattern” from cytogenetic bands 1p36.33 to 1p34.2 (molecular position: 849′466 and 42′465′700) indicating loss of heterozygosity (LOH) in the affected region in mosaic status (highlighted by green arrow) at MPAL stage highlighting a persisting significance of CSF3R activation at transformation. The chromosome 1 ideogram is shown in the bottom panel. The combination of deviating LOH pattern along with copy number of 2 defines copy neutral loss of heterozygosity (CN-LOH). Black arrow indicates the *CSF3R* locus at 1p34.3 (36′931′644–36′948′879), the vertical dotted blue line indicates the position of the centromere.

**Figure 3:**
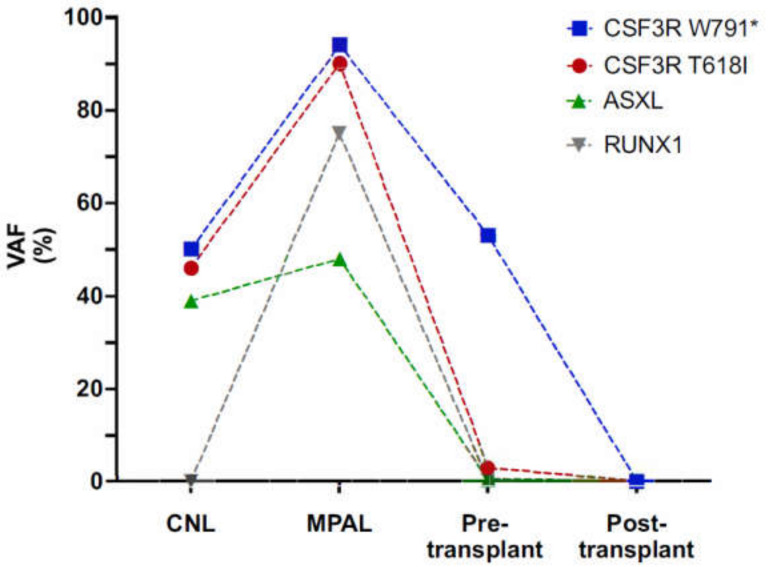
Clonal progression of *CSF3R* co-mutant CNL to acute leukemia of mixed phenotype (MPAL type) as reflected by sequential next generation sequencing. Upon CNL diagnosis, the germline *CSF3R*-W791* truncation mutation was complemented by a somatic *CSF3R*-T618I activating mutation at 46% variant allele frequency (VAF) along with an *ASXL1* C759fs mutation at 39% VAF. At transformation to mixed phenotype acute leukemia (MPAL), the *CSF3R* co-mutated clone had acquired an additional *RUNX1* R201Q mutation at 75% VAF. Mutant allele burden reflecting the MPAL clone was substantially reduced upon intensive induction therapy to *CSF3R* T618I 3% VAF, *ASXL1* C759fs 0.5% VAF and *RUNX1* R201Q 0.3% VAF before allogeneic transplantation and was undetectable after allogeneic hematopoietic stem cell transplantation.

To further evaluate the factors precipitating transformation in the *CSF3R* double mutant setting, we performed in-depth genetic analyses including NGS and SNP array karyotyping at several time-points during disease evolution. We observed copy neutral loss of heterozygosity (CN-LOH) at chromosome 1p at the time of transformation to acute leukemia (Figure 2), which substantiated the *CSF3R* co-mutated clone to variant allele frequencies >90% for both *CSF3R* mutations (Figure 3).

Also, the *ASXL1* mutant subclone expanded, and in addition a *RUNX1* R201Q mutation emerged with a high VAF of 75% relating to a mosaic trisomy 21 (Figure 3, Supplementary Figure S2). These findings suggest a persisting impact of CSF3R activation on transformation to acute leukemia, which is further complemented by additional high-risk mutations such as in the *RUNX1* gene.

## 4. Discussion

The discovery of *CSF3R* mutations in the majority of CNL patients has identified constitutive activation of hematopoietic cytokine receptor signaling as essential to induce neutrophilia in CNL. This is analogous to other myeloproliferative neoplasms (MPN) including polycythemia vera (PV), essential thrombocythemia (ET) and primary myelofibrosis (PMF) [2]. Experimental studies in murine models have validated *CSF3R* activating mutations such as T618I as driver mutations that promote development. *CSF3R*-T618I has become an established biomarker facilitating CNL diagnosis and also provides a therapeutic target for treatment with the JAK1/2 inhibitor ruxolitinib [4,5,6].

CNL is a rare hematologic disorder with variable clinical course, but dismal prognosis upon transformation [20]. Thus, a deeper understanding of the molecular mechanisms underlying pathogenesis and transformation is required. So far, transformation of CNL to acute leukemia has been reported to present as acute myeloid leukemia (AML) [21], and recently, a first case of CNL transforming to B-lymphoblastic leukemia has been described [22]. Here we investigated early onset CNL based on a patient presenting with CNL at a young age of 33 years, who showed progression to acute leukemia of mixed phenotype (MPAL), which to our knowledge represents the first report of such manifestation. As CNL is rare, characterization of additional individuals with transformation to acute leukemia of MPAL-type will be required to see whether mixed phenotype acute leukemia particularly associates with early onset CNL, and to confirm the importance of particular molecular alterations.

*CSF3R* truncation mutations in CNL primarily co-occur with auto-activating point mutations (such as T618I) and are rarely found in isolation [23]. While *CSF3R* truncation mutations promote leukemic transformation in severe congenital neutropenia (SCN), their significance for CNL pathogenesis has not been fully clarified. So far, *CSF3R* truncation mutations have not been reported in the germline. It is an intriguing question whether they would promote acquisition of additional, transformative *CSF3R* activating mutations or the expansion of clones with these mutations thus conferring predisposition states for CNL pathogenesis and progression. One case report on a child with a T618I germline mutation developing CNL with a somatic W791* mutation has been published [9]. To our knowledge, CNL based on germline *CSF3R* W791* along with an acquired T618I mutation has not been described thus far. Analyses of extensive family pedigrees would be required to assess leukemia predisposition based on CSF3R truncations, a resource which is unavailable.

The presence of both a *CSF3R* point and truncating mutation raises the question of whether they are on the same allele, and thus present in the same protein product. Previous sequencing of a very limited number of cases revealed that point and truncating mutations can occur on the same allele, but analysis of more cases is needed to know if they are usually present *in cis* [2]. Thus, we performed molecular analyses of the allelic localizations of the *CSF3R* truncation and activating mutations sequentially at the several stages of the disease. We investigated patient bone marrow derived DNA at CNL and MPAL stages and revealed localization of truncation and activating mutations on the same allele of the *CSF3R* gene. While this finding suggests a potential predisposition for CNL development and progression by the *CSF3R* W791* germline truncation mutation, more analogous cases will be required to corroborate such a pattern.

At both the initial diagnosis of CNL and subsequent progression to MPAL, a truncating mutation in the epigenetic regulator *ASXL1* was observed. *ASXL1* mutations are associated with the premalignant condition, clonal hematopoiesis, and are often thought to be early events in myeloid leukemogenesis [24]. Indeed, serial sequencing of samples from patients with CNL on ruxolitinib therapy revealed that the *CSF3R* mutant clone can shrink dramatically while the *ASXL1* mutant clone remains, indicating that *ASXL1* mutations were antecedent in those cases [25]. Interestingly, in this case, the *ASXL1* mutant clone has a lower VAF than both *CSF3R* mutations at all timepoints studied, consistent with it being a later genetic event. This case highlights the multiple potential paths to CNL biogenesis.

We therefore sought to understand the molecular changes that enable the clonal evolution of CNL progressing to acute leukemia of MPAL type. NGS profiling along with SNP array karyotyping at several stages of disease progression revealed that clonal evolution was not halted but continued despite intermittent targeted inhibition of JAK2 signaling via the JAK1/2 inhibitor ruxolitinib, as has been reported for the classical, Philadelphia chromosome-negative MPN [26]. CN-LOH at chromosome 1p encompassing the *CSF3R* gene locus resulted in doubled *CSF3R* mutant allele burden for the activating T618I as well as the W791* truncation mutation, thus further enhancing receptor activation and overexpression upon progression. At the pre-transplant timepoint in which there is a near elimination of the *CSF3R*-T618I, *ASXL1*-C759fs and *RUNX1*-R201Q mutant clone, the variant allele frequency of *CSF3R*-W791* returns to near 50% frequency, indicating that chemotherapy-induced clearance of the leukemic clone effectively restores the locus to heterozygosity (Figure 3). The persistence of the *CSF3R* W791* at 50% VAF pre-transplant, further supports the idea that this clone was germline. This finding highlights the persistent role of CSF3R activation in CNL, and at the stage of transformation to acute leukemia of MPAL type. It further underscores the need for therapeutic approaches to target mutational CSF3R activation.

In addition to CN-LOH at the *CSF3R* locus, transformation to MPAL was also associated with the acquisition of a point mutation in *RUNX1*, a transcription factor that regulates myeloid development. *RUNX1* mutations are associated with poor prognosis in AML [27,28]. *RUNX1* and *CSF3R* are frequently co-mutated in the progression to acute myeloid leukemia from SCN [18]. Neither acquisition of a *RUNX1* point mutation or CN-LOH at the *CSF3R* locus readily explain the mixed myeloid and lymphoid phenotype associated with transformation to acute leukemia.

MPAL is known to harbor various AML- and ALL-associated mutations. Characterization of the genetic basis of MPAL is still incomplete and a specific mutational pattern has not been consistently identified so far. Takahashi and colleagues [29], who described the genomic profiles of 13 adult patients with B/M MPAL, reported prevalent *ASXL1* mutations (23% of individuals) as well as *RUNX1* mutations (46% of individuals) similar to the mutational pattern in our MPAL patient. Other mutations known to be prevalent in hematological malignancies including *FLT3*, *NRAS* or *SRSF2* mutations have also been repeatedly reported in MPAL [30]. Of note, a landmark study by Alexander and colleagues, which analyzed mutational profiles of 35 patients, specifically identified *ZNF384* rearrangements at high incidence (43%) in children [31]. *CSF3R* mutations were found in two prior cases of early T-cell precursor acute lymphoblastic leukemia, in which the leukemic cells can also harbor both lymphoid and myeloid markers. However, in these cases the *CSF3R* mutations were accompanied by *NOTCH1* mutations which are potent lymphoid drivers [2,32]. It is possible that another unidentified mutation promotes the mixed leukemic phenotype in this case, or that alternately the germline nature of this mutation causes leukemia associated mutations to be present in a more primitive and multipotent stem cell than what is typically associated with CNL. The exact cell of origin for CNL is still unknown.

## 5. Conclusions

The molecular characterization of this early onset CNL provides new insights into CNL biogenesis and highlights essential open issues which require evaluation to further enhance our understanding of CNL development and progression to enable development of more effective therapeutic strategies. *CSF3R* truncation mutations were shown to be sensitive to dasatinib via Src kinase inhibition, but it is not known whether co-targeting *CSF3R* truncations such as W791* by dasatinib would provide therapeutic benefit in *CSF3R* co-mutant cases along with ruxolitinib treatment. Furthermore, the intriguing question whether dasatinib would be beneficial in a germline truncation situation to avoid progression to CNL and acute leukemia remains unanswered. Further molecular studies of additional patients with CSF3R driven malignancies will be essential to elucidate these theories, and to shed light on the complex evolution of CNL.

## Figures and Tables

**Table 1:** Pre-diagnostic, diagnostic and transformation phase of a patient with early onset CNL upon co-mutant CSF3R germline truncation and acquired activating mutation.

Variation	Phase	Treatment
Pre-diagnosis	Germline *CSF3R* truncation mutation W791*	none
Diagnosis	CNL	1st line: Hydroxyurea 2nd line: Ruxolitinib (progressive splenome-galy/neutrophilia) 3rd line: Pegylated interferon alpha (wish for children)
Transformation	Acute leukemia of MPAL type	Induction chemotherapy with Cytarabine + Idarubicin (1 cycle) followed by azacytidine Allogeneic HSCT from HLA-identical sibling after reduced intensity conditioning
Last follow-up	Complete molecular remission	None

CNL chronic neutrophilic leukemia, MPAL mixed phenotype acute leukemia, HSCT hematopoietic stem cell transplantation.

## Data Availability

The data presented in this study are available on request from the corresponding author (sara.meyer@unibas.ch). The data are not publicly available due to privacy/ethical reasons.

## Supplementary Materials

The following supporting information can be downloaded at: https://www.mdpi.com/article/10.3390/curroncol29020068/s1, Figure S1: Identification of germline origin of CSF3R-W791* but not T618I mutation, Figure S2: SNP array indicating mosaic trisomy 21 in the MPAL stage.
