# Supplementary material for: Co-Occurring CSF3R W791* Germline and Somatic T618I Driver Mutations Induce Early CNL and Clonal Progression to Mixed Phenotype Acute Leukemia
Source: Curr Oncol. 2022 Feb 1;29(2):805–15. doi: 10.3390/curroncol29020068 (PMC8870680; doi:10.3390/curroncol29020068)
Supplement: Supplementary file 1 [file curroncol-29-00068-s001.zip › curroncol-1560028-supplementary.pdf]

Current Oncology

Supplementary Material

Co-Occurring CSF3R W791* Germline and Somatic T618I Driver Mutations Induce Early CNL and Clonal Progression to Mixed Phenotype Acute Leukemia

Franziska C. Adam [1,†], Jakub Szybinski [2,†], Joerg P. Halter [1], Nathan Cantoni [3], Friedel Wenzel [4], Katharina Leonards [1], Sime Brkic[2], Jakob R. Passweg [1], Ivo Touw [5], Julia E. Maxson [6] and Sara C. Meyer [1,2,*]

[1] Division of Hematology, University Hospital Basel, Basel, Switzerland
[2] Department of Biomedicine, University Hospital Basel and University of Basel, Basel, Switzerland
[3] Kantonsspital Aarau, Aarau, Switzerland
[4] Institute of Medical Genetics and Pathology, University of Basel, Switzerland
[5] Department of Hematology, Erasmus University Medical Center, Rotterdam, The Netherlands
[6] Knight Cancer Institute, Division of Oncologic Sciences, Oregon Health & Science University, Portland, OR, USA.
* Correspondence: sara.meyer@unibas.ch; Tel.: +41 61 556 59 65
† These two authors contributed equally (alphabetical order).

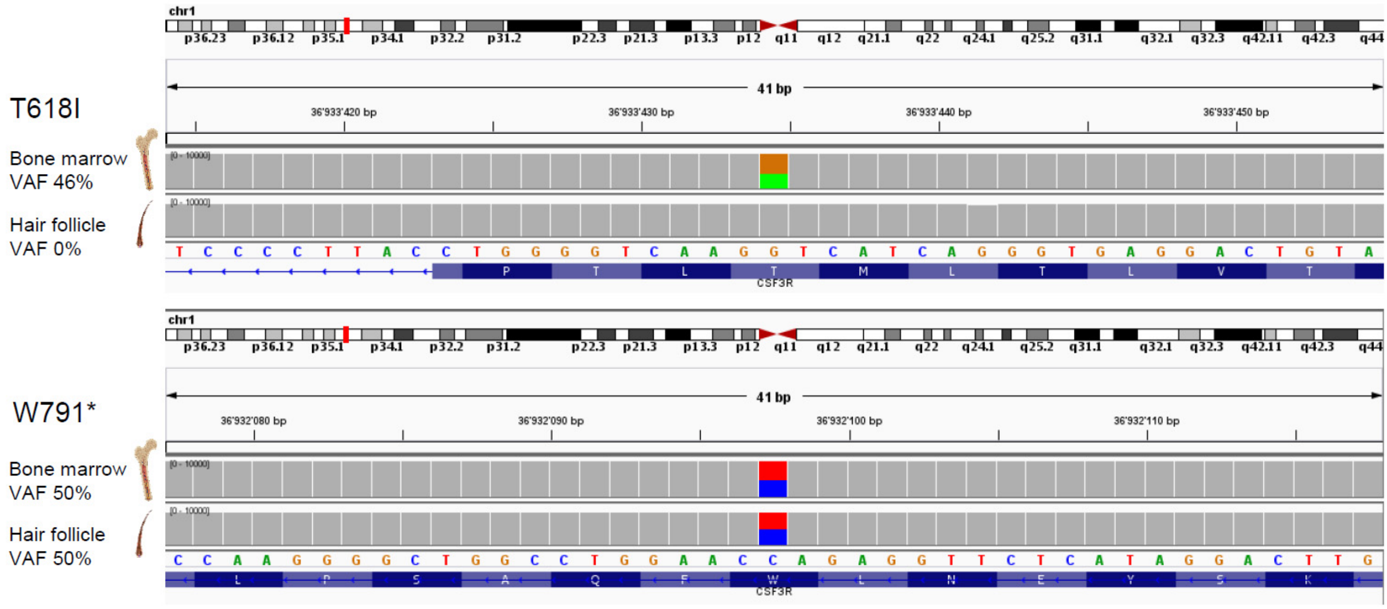

Figure S1. Identification of germline origin of *CSF3R*-W791* but not T618I mutation. By next generation sequencing of a 39 myeloid gene panel in DNA isolated from bone marrow and hair follicle, the *CSF3R*-W791* mutation was present both in bone marrow (BM) and hair follicle while T618I was detected in BM but not in hair follicle.

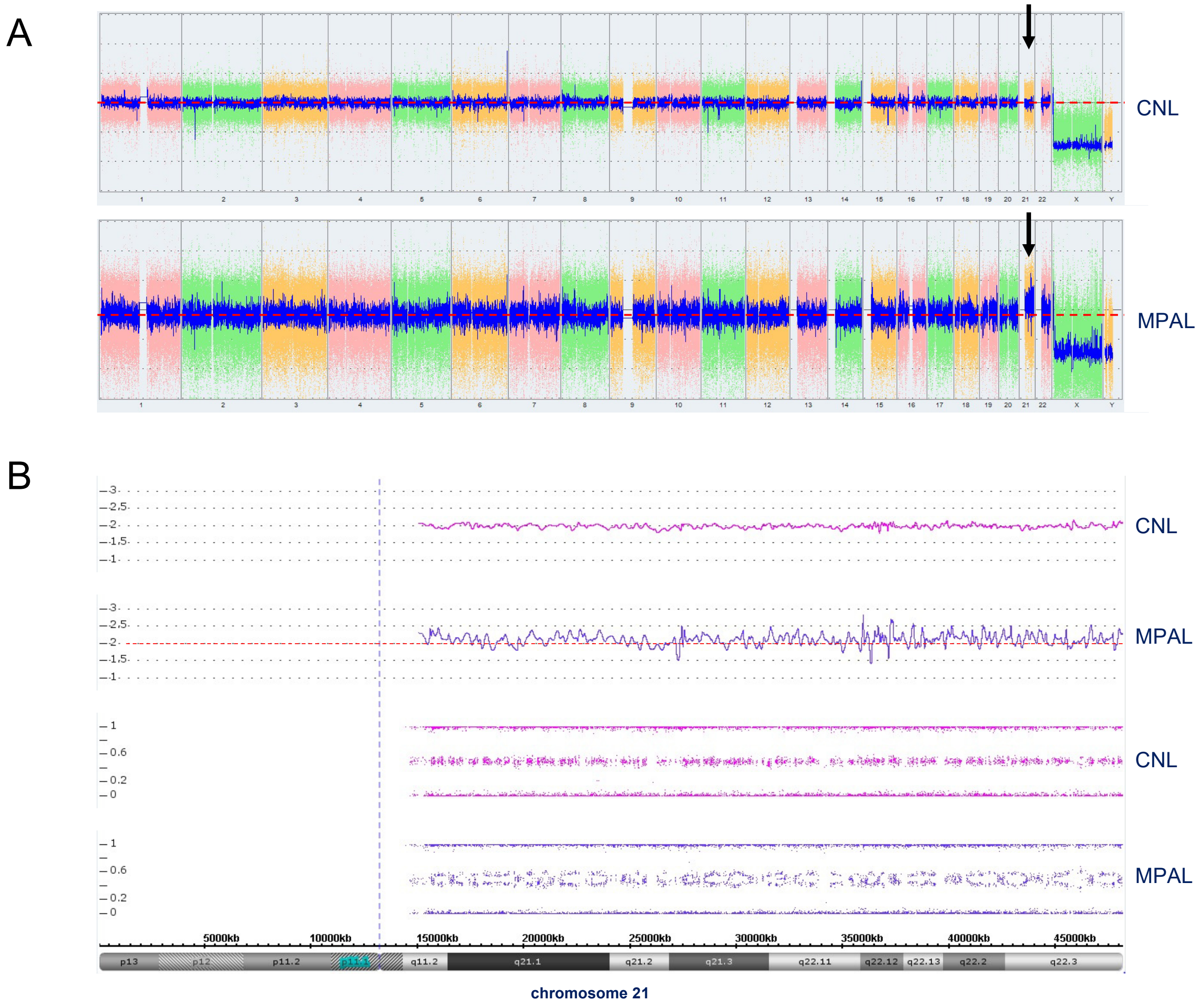

Figure S2. SNP array indicating mosaic trisomy 21 in the MPAL stage. **A.** Whole genome views of SNP array at CNL stage (top) and at MPAL stage (bottom) indicate a subtle increase of copy number at chromosome 21 in the MPAL stage (black arrows). Dotted red line indicates copy number 2. **B.** Detailed representation of chromosome 21. Pink color represents DNA from CNL stage, blue color represents DNA from MPAL stage. Upper two panels show smooth signal distribution for DNA from CNL with unchanged copy number of 2, whereas smooth signal distribution for DNA from MPAL stage is slightly increased above copy number of 2 (dotted red line). Middle panels show B-allele frequency (BAF) distributions with DNA from CNL stage (pink) with normal "three-line pattern" indicating normal allele distribution, and DNA from MPAL stage (blue) with "four-line pattern" indicating a change in allele distribution. The bottom panel shows the chromosome 21 ideogram. The vertical dotted blue line indicates the position of the centromere. The specific combination of deviating allele pattern and increased copy number reflects mosaic trisomy 21 status consistent with variant allele frequency of >50% of the *RUNX1* mutation at MPAL stage.
